# Characteristics and Prognosis of Autoimmune Encephalitis in the East of China: A Multi-Center Study

**DOI:** 10.3389/fneur.2021.642078

**Published:** 2021-05-31

**Authors:** Shan Qiao, Huai-kuan Wu, Ling-ling Liu, Ran-ran Zhang, Mei-ling Wang, Tao Han, Shan-chao Zhang, Xue-wu Liu

**Affiliations:** ^1^Department of Medical Genetics, School of Basic Medical Sciences, Cheeloo College of Medicine, Shandong University, Jinan, China; ^2^Department of Neurology, The First Affiliated Hospital of Shandong First Medical University and Shandong Provincial Qianfoshan Hospital, Jinan, China; ^3^Department of Neurology, Liaocheng People's Hospital, Liaocheng, China; ^4^Department of Neurology, Qilu Hospital, Cheeloo College of Medicine, Shandong University, Jinan, China; ^5^Department of Neurology, Binzhou Medical University Hospital, Binzhou, China; ^6^Department of Neurology, Shandong Provincial Hospital, Shandong University, Jinan, China; ^7^Department of Oncology, Jinan Central Hospital Affiliated to Shandong University, Jinan, China; ^8^Institute of Epilepsy, Shandong University, Jinan, China

**Keywords:** autoimmune encephalitis, epidemiology, clinical features, prognosis, relapse

## Abstract

**Objective:** This study aimed to investigate epidemiological characteristics, clinical manifestations, and long-term outcomes of patients with autoimmune encephalitis (AE) in the east of China.

**Methods:** From January 2015 to December 2019, 226 potential AE patients were recruited from five clinical centers, and a total of 185 patients who met the diagnostic criteria were included in the study. We retrospectively reviewed clinical features, auxiliary examinations, details of treatments, and outcomes of AE, and identified risk factors of poor prognosis. Modified Rankin Scale scores were used to evaluate neurological function, and scores of 3–6 indicated a poor-prognosis.

**Results:** Patients with five main subtypes of AE were enrolled in the study, as follows: anti-NMDAR (79), anti-LGI1 (55), anti-CASPR2 (30), anti-GABABR (16), and anti-AMPAR (5). Among 185 patients, 58.38% (108/185) were male and 41.62% (77/185) were female. The median age at disease onset was 41 years (interquartile range, 17–62). The most common clinical manifestations of AE were seizures (146, 78.92%) and memory deficit (123, 66.49%). A total of 95 (51.35%) patients had abnormal brain magnetic resonance imaging results. Electroencephalographic findings were abnormal in 131 (70.81%) patients, and 168 (90.81%) and 26 (14.05%) patients were treated with first- and second-line immunotherapies, respectively. All surviving patients were followed-up for at least 1 year (range 12–36 months). Good clinical outcomes were achieved in 117 (63.24%), while 68 (36.76%) patients had a poor prognosis. Further, 33 (17.84%) patients relapsed and 10 (5.41%) died within 1 year post-discharge. Older patients tended to have a poorer prognosis, and the occurrence of mental behavioral disorders, movement disorders, disturbance of consciousness, central hypoventilation, and tumors were overrepresented in the poor-prognosis group.

**Conclusions:** AE is a treatable disease, and most patients have a good prognosis. There are differences in the clinical manifestations of patients with different AE subtypes. Some with AE will relapse, and long-term follow-up is of great significance for further research.

## Introduction

Autoimmune encephalitis (AE) is a class of severe brain inflammatory diseases that are mediated by autoimmune mechanisms, mostly with acute or subacute seizures, cognitive impairment and mental symptoms as main clinical manifestations. The AE class accounts for about 10–20% of encephalitis cases (1). AE is thought to be associated with antibodies to neuronal cell-surface proteins such as N-methyl-D-aspartate receptor (NMDAR), leucine-rich glioma-inactivated 1 (LGI1), contactin-associated protein-like 2 (CASPR2), gamma-aminobutyric-acid B receptor (GABABR), etc. Some differences have been observed with regard to the clinical manifestations and prognosis of patients with different subtypes of AE (2, 3). Most AE patients respond well to immunotherapy. However, some types of encephalitis have a poor prognosis, and often produce symptoms such as intractable seizures and cognitive dysfunction.

In the past 10 years, with the advancement of diagnostic technology, the antibody spectrum of AE has continued to expand, and research on its diagnosis and treatment has also continued to progress (4–6). Disease incidence, clinical features of disease, and strategies used to treat AE patients in different countries and regions differ. In China, a consensus opinion for improving the identification and management of AE patient was proposed 2017 (4). Due to the low incidence of AE, especially rare LGI1, GABAR, and anti-a-amino-3-hydroxy-5-methyl-4-isoxazolepropionic acid receptor (AMPAR) encephalitis classes, clinical studies with large sample sizes are rare, both in China and abroad (7–9). At present, the efficacy of immunotherapy and factors associated with a poor patient prognosis have not been determined. In addition, existing criteria for AE rely on antibody testing to some extent, therefore, delays in the diagnosis of patients, and failure to diagnose antibody-negative patients may occur. According to previous studies, early use of immunotherapy is closely related to AE prognosis (10, 11). Therefore, research on the clinical characteristics of AE, and the diagnosis and prognostic factors of the disease using a large sample size that includes multiple AE subtypes is important.

To improve our understanding of different subtypes of AE and to provide knowledge that may improve its clinical diagnosis and treatment, we performed a retrospective analysis of the clinical characteristics, treatment, outcomes, and prognostic factors of 185 AE patients treated in multiple clinical centers in China.

## Methods

### Study Design and Population

This retrospective study included patients who were serum- and/or cerebrospinal fluid (CSF)-positive for neuron surface antibodies who were diagnosed with AE according to published diagnostic criteria between January 2015 and December 2019. Patients were recruited from the following 5 clinical centers: Qilu Hospital, Cheeloo College of Medicine, Shandong University; The First Affiliated Hospital of Shandong First Medical University; Shandong Provincial Hospital, affiliated with Shandong University; Affiliated Hospital of Binzhou Medical College; and Liao Cheng People's Hospital.

The inclusion criteria were as follows: (1) acute or subacute onset of 1 or more clinical manifestations of AE, including seizures, memory deficit, psychosis, and speech disturbance related to the limbic system; (2) testing serum- and/or CSF-positive for neuron surface antibodies; and (3) reasonable exclusion of other disorders. The exclusion criteria included incomplete clinical data and negative serum and CSF antibody tests. This study was conducted in accordance with the Declaration of Helsinki and was approved by the Ethics Committee of Qilu Hospital of Shandong University (approval number: KYLL-202008-044). Written informed consent was obtained from all study participants and their legal guardians.

### Data Collection

Clinical data, results of cerebrospinal fluid (CSF) and serum analyses, electroencephalographic (EEG) and brain magnetic resonance imaging (MRI) findings, details of treatments, and patient prognosis were recorded. Serum and CSF samples from all 5 medical centers were sent to the same testing center for evaluation. Autoantibodies to NMDAR, LGI1, CASPR2, GABABR, AMPA1, AMPA2 were assessed for all 226 patients via an indirect immunofluorescence protocol that was performed in accordance with the manufacturer's instructions (Euroimmun, Germany). A cell-based assay with high specificity and sensitivity was used to analyze CSF and serum samples from each patient. The initial dilution titers of CSF and serum were 1:1 and 1:10, respectively. Serum antibody titers were considered weakly positive at 1:10, positive at 1:32–1:100, and strongly positive at 1:320. The titers of CSF antibodies were considered weakly positive at 1:1, positive at 1:3.2–1:10, and strongly positive at 1:32 or above.

### Assessment of AE Prognosis

All patients were followed up after being discharged from the hospital to assess AE prognosis. During the follow-up, two experienced doctors used modified Rankin Scale (mRS) scores to assess effects of treatments and clinical outcomes. According to the mRS score at discharge, the patients were divided into two groups, as follows: patients with a mRS score ≤ 2 were placed in the “good-prognosis” group; and patients with a mRS score > 2 were placed in the “poor-prognosis” group. Patients were followed-up every 3 months throughout the 1st year post-discharge, and every 6 months thereafter. All patients were followed up for at least 1 year.

### Statistical Analysis

Statistical analyses were performed using SPSS IBM 25.0 and GraphPad Prism 8.0 software. Categorical variables were described as percentages; continuous variables distributed normally were presented as means and standard deviations. Non-normal data were presented as a median and interquartile range (IQR). The Student's *t*-test was used to compare the mRS scores before and after treatment. Symptoms and demographic data were analyzed using the χ^2^ test or Fisher exact test for categorical variables, and the Mann-Whitney *U*-test for continuous variables. Values of *p* < 0.05 were considered significant.

## Results

### Baseline Demographic Trends Associated With Disease Incidence

**Figure 1:**
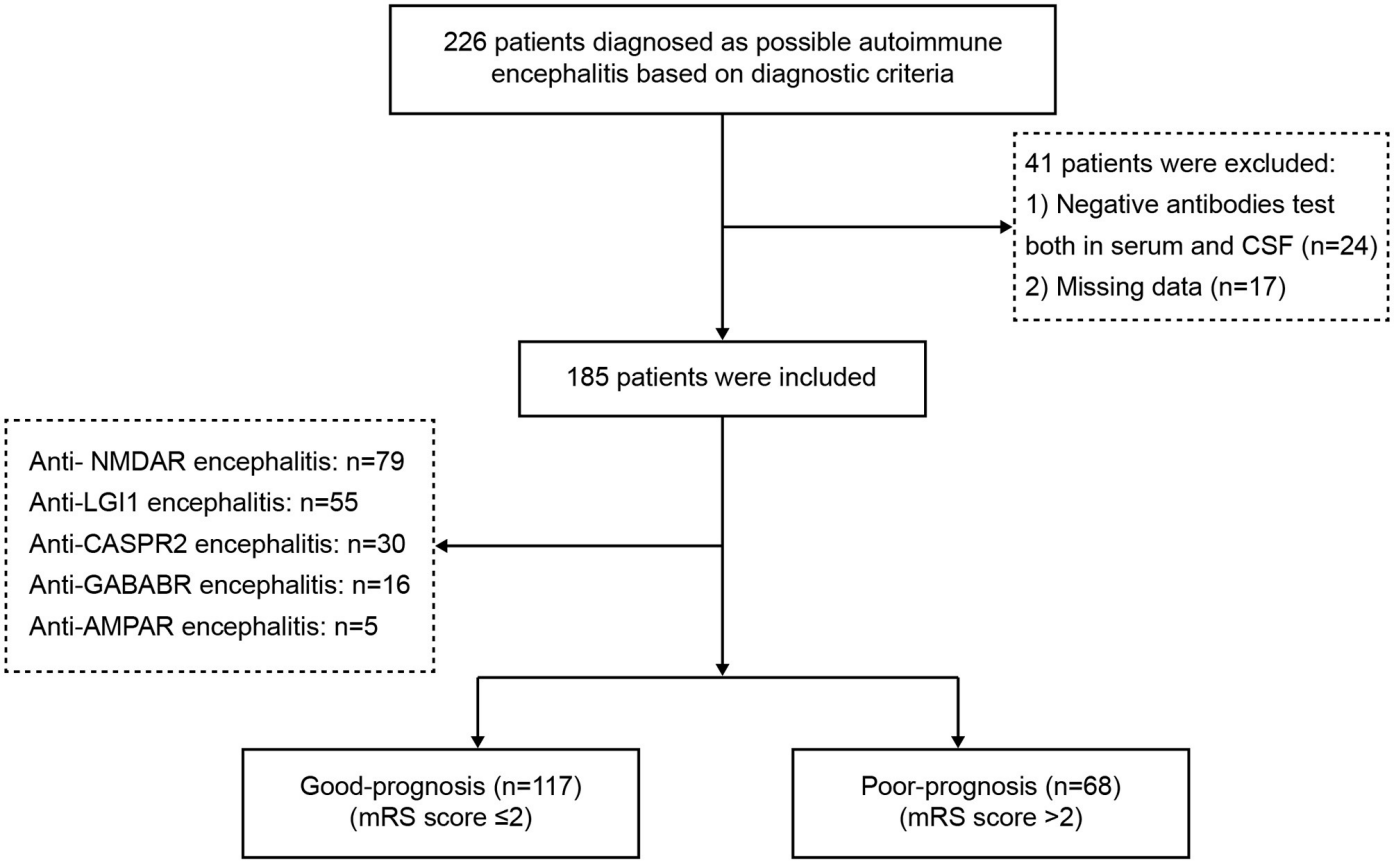
The flowchart of patient selection. mRS, Modified Rankin Scale (mRS).

**Figure 2:**
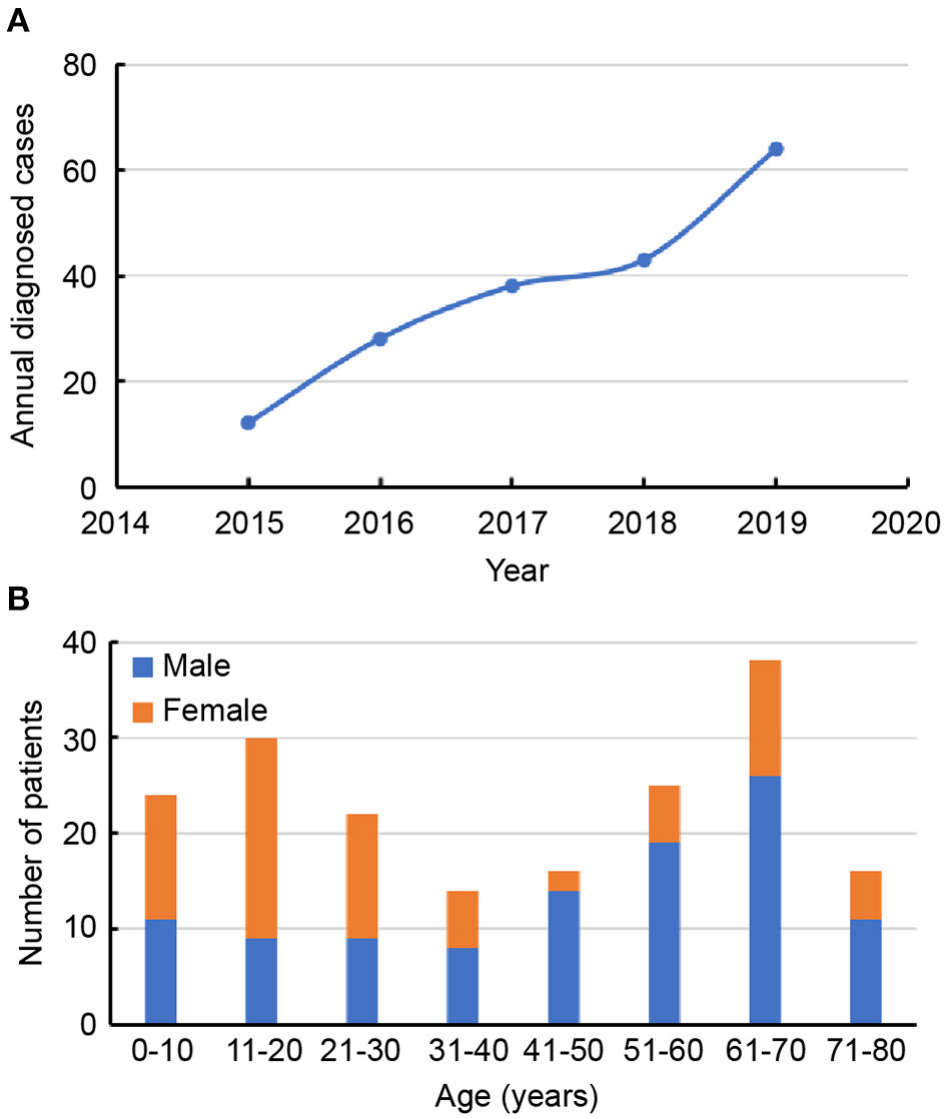
**(A)** Trends in the numbers of annual diagnosed cases of autoimmune encephalitis. **(B)** Distribution of gender and age of patients with autoimmune encephalitis.

From January 2015 to December 2019, 226 potential AE patients were recruited for inclusion in the study. After exclusion criteria were applied, a total of 185 patients with AE remained, including 79 patients with anti-NMDAR encephalitis, 55 with anti-LGI1 encephalitis, 30 with anti-CASPR2 encephalitis, 16 with anti-GABABR encephalitis, and 5 with anti-AMPAR encephalitis. In Figure 1, a flowchart that describes patient selection is shown. In the study, the annual number of confirmed cases trended upward (Figure 2A). Among the 185 patients included in the study, 58.38% (108/185) were male and 41.62% (77/185) were female. The median age of AE onset of included patients was 41 years (IQR, 17–62). It is worth noting that peak frequencies of AE onset tended to occur at ages 11–20 and 61–70 (Figure 2B).

All patients showed acute or subacute onset of disease, and 47 (25.41%) patients exhibited prodromal symptoms such as headache, fever, or other symptoms of non-specific upper respiratory tract infection. The average time from symptom onset until diagnosis was 6.9 (IQR, 3.5–27) weeks. In most patients, initial symptoms included seizures (48.64%), memory deficit (22.70%), and mental behavioral disorders (19.46%). The remaining 9.19% of patients experienced sleep, speech, and movement disorders, and autonomic symptoms at disease onset (Table 1).

**Table 1:** Clinical features of patients (*n* = 185).

**Characteristics**	**Values**
Sex, male/female	108/77
Age at onset, y, median, IQR	41 (17–62)
Time from symptom onset until diagnosis, week, median, IQR	6.9 (3.5–27)
Prodromal symptoms, n (%)	47 (25.41)
**Initial symptoms, n (%)**	
Seizures	90 (48.64)
Memory deficit	42 (22.70)
Mental behavioral disorders	36 (19.46)
Others	17 (9.19)
**Clinical syndrome, n (%)**	
Seizures	146 (78.92)
Memory deficit	123 (66.49)
Mental behavioral disorders	110 (59.46)
Disturbance of consciousness	69 (37.30)
Sleep disorder	67 (36.22)
Speech disorders	64 (34.59)
Movement disorder	58 (31.35)
Headache	24 (12.97)
Autonomic dysfunction	29 (15.68)
Fever	28 (15.14)
Involuntary movements	27 (14.60)
Complicated with tumors	20 (10.81)
Central hypoventilation	17 (9.19)
Admission to ICU	13 (7.03)
**Treatment**	
First-line immunotherapy, n (%)	168 (90.81)
Second-line immunotherapy, n (%)	26 (14.05)
**Prognosis**	
Good-prognosis	117 (63.24)
Poor-prognosis	68 (36.76)
Relapse	33 (17.84)
Mortality	10 (5.41)

### Clinical Characteristics of AE

The most common clinical manifestations of AE were seizures (146, 78.92%), memory deficit (123, 66.49%) and mental behavioral disorders (10, 59.46%). Table 1 describes the clinical characteristics of the 185 patients included in the study in detail. Among these patients, 20 (10.81%) also had tumors. Patients with anti-NMDAR encephalitis have a higher incidence of ovarian teratoma than other AE class, and common tumor types in patients with anti-GABAR encephalitis are small cell lung cancer and mediastinal. In all 55 patients with anti-LGI1 encephalitis, no tumors were observed. During the course of the disease, 17 (9.19%) patients had central hypoventilation, and 13 (7.03%) were treated in an intensive care unit due to respiratory failure and status epilepticus (SE).

### Auxiliary Examinations

**Figure 3:**
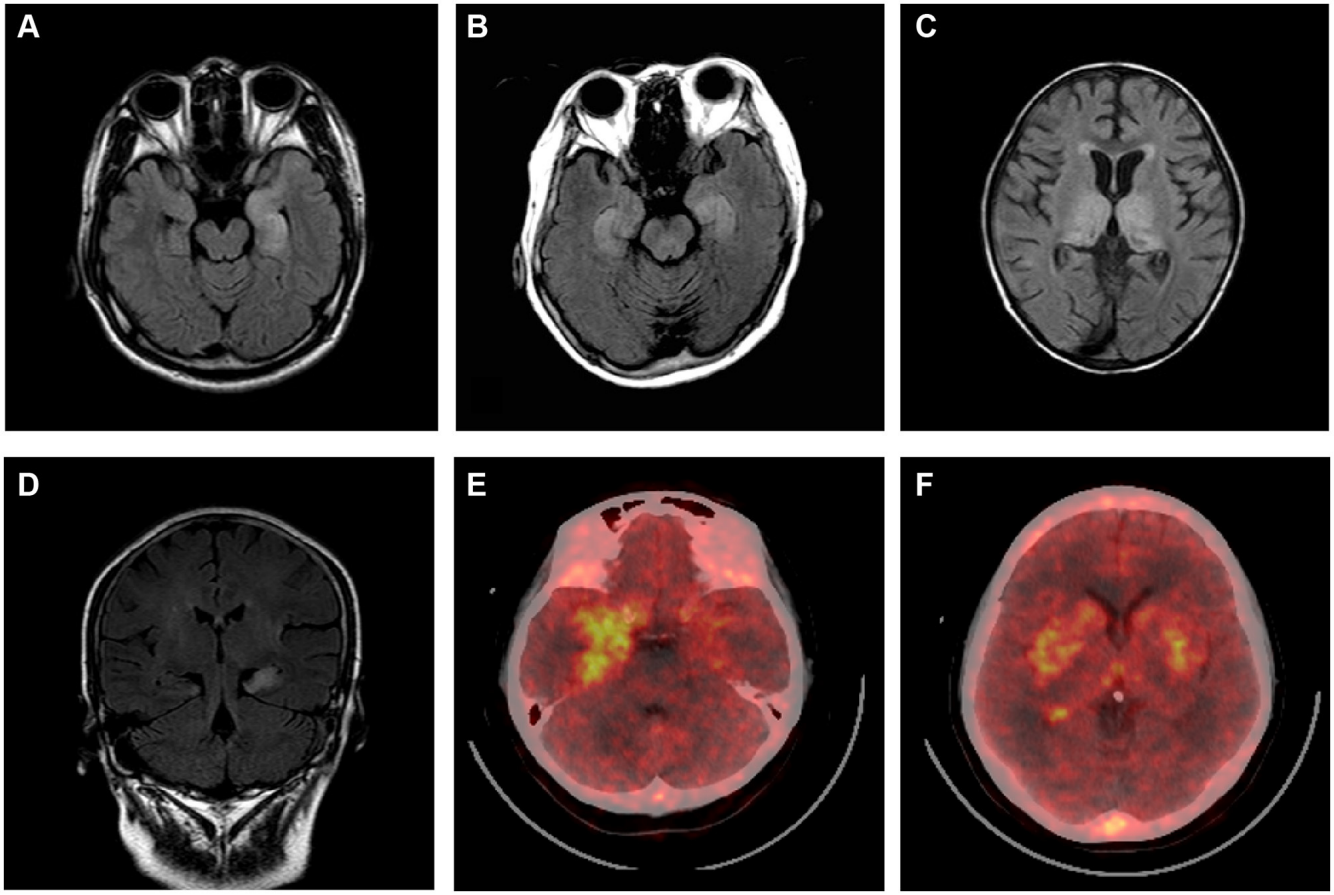
Brain MRI and positron emission tomography (PET) scans of patients with autoimmune encephalitis. Brain MRI T2-flair images **(A–D)**. Bilateral high signal in the temporal lobe **(A,B,D)**. Bilateral abnormal signals in the medial temporal lobe and occipital lobe **(C)**. PET images show temporal cortex (more significant on the right) **(E)** and bilateral hypermetabolism in the basal ganglia **(F)**.

Brain MRI, EEG, and CSF and serum antibody findings of 185 patients are described in the study (Table 2). A total of 95 (51.35%) patients had abnormal brain MRI results. The main lesions observed occurred in the temporal (61, 32.97%), and frontal (12, 6.48%) lobes. Other abnormal brain MRI findings revealed lesions located in the hippocampus, basal ganglia, occipital lobes, insular lobes, cerebellum, cortex, and white matter. Two patients underwent brain positron emission tomography examinations while in the acute phase of disease: 1 patient had hypermetabolism within the bilateral temporal lobe, and the other patient experienced bilateral hypermetabolism of the basal ganglia and temporal lobe. EEG findings of 131 (70.81%) patients were abnormal, with 84 (45.41%) cases involving unilateral or bilateral non-specific slow waves, and 47 (25.41%) cases of epileptiform discharges (such as sharp waves, spike waves, sharp slow wave complexes, or spike slow wave complexes). Typical imaging findings of patients included in the study are shown in Figure 3. CSF findings revealed that 57 (42%) patients had elevated total protein concentrations, and 19 (10.27%) cases were positive for oligoclonal bands. Serum and CSF antibody titers of the 185 included patients ranged from 1:10 to 1:320, and 1:1 to 1:300, respectively. Of all patients considered, 143 patients were serum and CSF positive for AE antibodies, 18 were exclusively CSF positive for AE antibodies, and 31 were exclusively serum positive for AE antibodies. Details of serum and CSF antibody tests are shown in Table 3. Serum analysis showed that 55 (29.73%) patients had hyponatremia and 34 (18.38%) had hypokalemia. Among the 185 patients considered, 63 (34.05%) displayed abnormal thyroid function.

**Table 2:** Summary of the main ancillary tests results (*n* = 185).

**Brain MRI**		**n (%)**
Total with abnormal findings		95 (51.35)
Temporal lobe		61 (32.97)
Frontal lobe		12 (6.48)
Basal ganglia		9 (4.86)
Others		13 (7.03)
**EEG**		
Total with abnormal findings		131 (70.81)
Epileptic discharges		47 (25.41)
Slow waves		84(45.41)
**CSF analysis**	**Median (range)**	**n (%)**
Opening pressure (mm H_2_O)	150 (80–300)	
WBC (×10^6^/L)	16.0 (1.0–92.0)	
Protein (g/L)	0.65 (0.06–1.16)	
Glucose (mmol/L)	4.58 (3.02–6.50)	
Chloride (mmol/L)	127 (105–134)	
Positive oligoclonal bands		19 (10.27)
**Serum analysis**		
Hyponatremia		55 (29.73)
hypokalemia		34 (18.38)
Abnormal thyroid function		63 (34.05)

**Table 3:** Details of antibody titers in the CSF and serum of antibody-positive patients.

		**Negative**	**Weakly positive**	**Positive**	**Strongly positive**
NMDAR	Serum	20	21	26	4
	CSF	8	46	25	2
LGI1	Serum	3	4	41	1
	CSF	7	24	5	1
CASPR2	Serum	8	9	11	1
	CSF	6	7	2	1
GABAR	Serum	0	6	6	4
	CSF	2	6	6	0
AMPAR	Serum	0	1	4	0
	CSF	1	2	2	0

### Treatments and Outcomes

**Figure 4:**
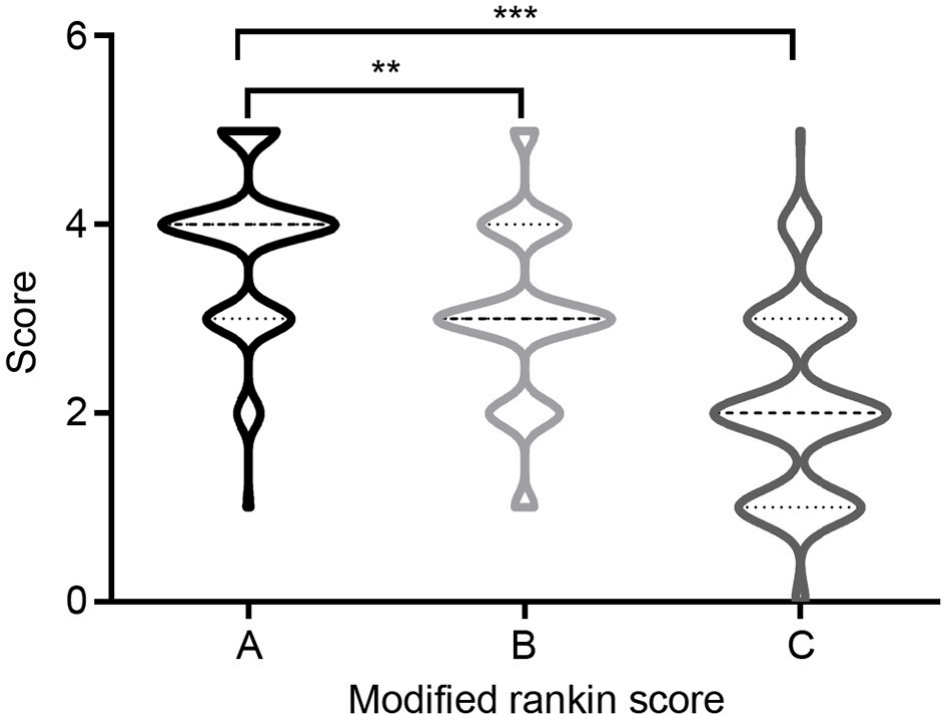
Modified Rankin Scale (mRS) at onset and follow-up. **(A)** mRS at onset; **(B)** mRS at discharge; **(C)** mRS at 12-months follow-up. **P < 0.01, ***P < 0.001.

Overall, 168 (90.81%) patients received first-line immunotherapy, and 128 patients (69.17%) received a combined regimen of repeated steroid and intravenous immunoglobulin (IVIG) administration. A total of 161 (87.03%) patients received steroids, of whom 109 (58.92%) received high-dose methylprednisolone intravenous drip treatment (with an initial dose of 1,000 or 500 mg methylprednisolone). IVIG was administered to 135 (72.98%) patients, and seven were treated with IVIG alone. Most patients responded well to first-line immunotherapy, and mRS scores after immunotherapy were significantly lower those determined at disease onset (Figure 4). Second-line immunotherapy (26, 14.05%) was administered to a small proportion of the patients considered, usually due to its cost, or concerns regarding its side effects. Second-line immunotherapy drugs used included rituximab, mycophenolate mofetil, cyclophosphamide and azathioprine. These therapies were used to treat patients who experienced severe or refractory acute phase disease, or those who had relapsed. In our cohort, 33 (17.84%) patients experienced disease relapse, and 10 (5.41%) patients died of severe lung infections, SE, tumors and other serious complications within 1 year after discharge.

All surviving patients were followed-up for at least a year post-discharge (range 12–57 months). At the 12-month follow-up assessment, 117 (63.24%) patients had attained satisfactory clinical outcomes (good prognosis), while clinical outcomes for 68 (36.76%) patients were poor (poor prognosis). The median mRS score at the last follow-up was 2 (IQR 1–3), which was significantly lower than the score of 4 (IQR 3–4) determined at disease onset (*p* < 0.0001). Univariate analysis indicated that there were significant differences between the good- and poor-prognosis groups with regard to clinical manifestation and auxiliary examination values. Table 4 summarizes comparisons that were made between groups of patients with good and poor clinical outcomes. The ages of included patients at AE onset ranged from 1 to 77 years, with a median age of 41 years (IQR, 17–62). There was a significant difference between the mean age at AE onset of good- and poor-prognosis groups (*p* = 0.043). Notably, rates of mental behavioral disorders, movement disorder, disturbance of consciousness, central hypoventilation, and tumor occurrence of the poor-prognosis group were elevated relative to the good-prognosis group. In addition, the proportions of CSF positive-oligoclonal bands, hyponatremia and brain MRI abnormal signals were significantly higher in the poor-prognosis group than in good-prognosis group. Steroids and IVIG combined immunotherapy tended to result in better prognoses than other therapies (*p* = 0.011) (Table 4).

**Table 4:** Results from the univariate analysis of clinical data of two groups.

**Variable**	**Good-prognosis (*n* = 117)**	**Poor-prognosis (*n* = 68)**	**Statistical value**	***p***
Age (y)	23 (14–32)	29 (21–37)	−2.17	0.043^†^
**Clinical symptoms (n, %)**				
Seizures	93 (75.0%)	53 (86.9%)	3.472	0.054
Mental behavioral disorders	59 (47.6%)	51 (83.6%)	22.096	0.012^*^
Memory deficit	78 (62.9%)	45 (73.8%)	2.167	0.095
Movement disorder	27 (21.8%)	31 (50.8%)	16.027	0.037^*^
Disturbance of consciousness	30 (24.2%)	39 (63.9%)	27.612	<0.001^*^
Central hypoventilation	5 (4.0%)	12 (19.7%)	11.985	0.001^*^
Complicated with tumors	4 (3.2%)	16 (26.2%)	22.439	<0.001^*^
Admission to ICU	3 (2.4%)	10 (16.4%)	12.221	<0.001^*^
**CSF analysis**				
Increased level of protein	37 (29.8%)	20 (32.8%)	0.167	0.736
Positive oligoclonal bands	6 (4.8%)	13 (21.3%)	12.039	0.001^*^
**Serum analysis**				
Hyponatremia	48 (38.7%)	7 (11.5%)	14.516	0.001^*^
Abnormal thyroid function	43 (34.7%)	20 (32.8%)	0.065	0.870
Abnormal brain MRI signal	55 (44.4%)	40 (65.6%)	7.369	0.008^*^
Abnormal EEG findings	90 (72.6%)	41 (67.2%)	0.570	0.592
**Immunotherapy (n, %)**				
Steroids	27 (21.8%)	6 (9.8%)	3.976	0.065
IVIG	4 (3.2%)	3 (4.9%)	0.322	0.686
Steroids + IVIG	78 (62.9%)	50 (82.0%)	6.971	0.011^*^
Second-line treatment	14 (11.3%)	12 (19.7%)	2.378	0.176

† Z-value in the Mann-Whitney U-test; *χ^2^,

* *p-value in Fisher's exact test*.

### Relapse

In patients with AE who experienced clinical symptom improvement or stabilization for more than 2 months, the reappearance of symptoms, or aggravation of original symptoms (increased mRS score of 1 point or more) indicated the occurrence of relapse. During the follow-up period, 33 (17.84%) patients experienced a relapse, 19 with anti-NMDAR encephalitis, 7 with anti-LGI1 encephalitis, 3 with anti-CASPR2 encephalitis, 3 with anti-GABABR encephalitis, and 1 with anti-AMPAR encephalitis. The average time to relapse was 5.2 months (IQR, 4.6–10.7), and 27 of the 33 patients who experienced relapse did so within the 1st year of follow-up. Of all 33 patients who experienced relapse, 25 had abnormal brain MRI signals at disease onset, and the remaining 8 AE patients had no obvious brain MRI abnormalities initially or during disease recurrence.

## Discussion

In recent years, the incidence of AE has been increasing. However, the etiology and pathogenesis of AE remains unclear (12–14). Given that AE is a serious but treatable disease, the ability to perform a timely and accurate assessment of disease prognosis is important for providing appropriate therapy and improving clinical outcomes of AE. In this study, we retrospectively analyzed clinical characteristics, treatments, and clinical outcomes of 185 Chinese patients with AE from five clinical centers. Moreover, we preliminarily evaluated factors which are associated with a poor patient prognosis.

In this study, 108 (58.38%) male patients with AE were assessed, and the incidence rate of disease in males was higher than that of females. Anti-NMDA encephalitis was the most commonly observed AE type, followed by LGI1 encephalitis. The gender distribution and the proportion of AE subtypes tended to be consistent with those of previous reports (1, 15). In this study, the incidence of disease onset peaked in patients aged 11–20 and 61–70 years, a finding that differed from prior reports, which showed that peak incidence of disease onset occurred in young and middle aged patients (3, 14). The difference observed may be due to regional differences, including differing occurrence rates of AE subtypes in patient populations considered. This also suggests that differing age distributions of AE onset may occur in patients with different AE subtypes. The possibility of AE should be considered when elderly patients have acute or subacute mental disorders, sleep disorders and seizures.

In recent years, the diagnosis and treatment of AE have been increasingly studied, but the etiology and pathogenesis of AE have not yet been elucidated (5). Current research suggests that infections, tumors, etc. may be the cause of AE (16). In particular, infection is considered an important trigger of AE. AE development after infection with herpes simplex virus (HSV), cytomegalovirus, and rubella virus has been reported previously. Neuronal antibodies are frequently detected in patients with post-HSV encephalitis (HSE) autoimmune encephalitis (17, 18). Studies suggest up to 27% of post-HSE patients subsequently develop AE, and antibodies to the NMDAR are the most prevalent in these patients (9). In our cohort, 47 (25.41%) patients had infection symptoms, and 21 patients had herpes simplex virus IgG or/and cytomegalovirus IgG (data not shown). Similar to previous reports, infection symptoms were most common in patients with anti-NMDAR AE. Taken together, these findings support prior evidence indicating that AE may be induced by infection, and also provides ideas for further exploring the pathogenesis of AE.

Seizures, memory deficit, and mental behavioral disorders were the main clinical manifestations of AE in patients included in our cohort. Among these, the prevalence of seizures in anti-GABABR antibody encephalitis was 100%. In these patients, the effect of treatment with anti-epileptic drugs was not good, and we observed that immunotherapy must be provided in a timely manner, which is consistent previously reported findings (14, 19, 20). Most patients included in this study responded well to immunotherapy, and findings suggested that patients treated with steroids in combination with immunoglobulin tended to have a good prognosis. A small number of patients received second-line treatment, including those with unsatisfactory or relapsed after receiving first-line immunotherapy. Previous studies have suggested that the onset of anti-NMDAR encephalitis is closely related to B cell immunity, and the therapeutic mechanism of rituximab depends on the depletion of B cells (21, 22). However, only sporadic case reports support these findings, and no large-scale studies have been conducted. Therefore, the efficacy of second-line drugs such as rituximab requires further clinical verification. For patients whose first-line drug treatment is not effective, second-line drugs are options.

At present, the diagnosis of AE mainly relies on observing clinical manifestations and detecting neuronal antibodies (2, 23, 24). In the initial 226 patients with suspected AE who recruited according to diagnostic criteria, 24 patients were negative for serum and CSF neuron surface antibodies, and 186 patients were serum and/or CSF positive for the antibodies. This suggests that reliance on antibody testing for diagnosing AE may cause AE patients who are antibody-negative to be overlooked. Therefore, it is of great significance to explore alternatives for performing early-stage diagnosis of AE. In 2016, Graus et al. published diagnostic criteria for autoimmune limbic encephalitis in *Lancet Neurology*, which did not rely on the detection of antibodies (23). This diagnostic standard is widely used abroad. Although it simplifies diagnostic steps, it can improve the efficiency of early AE diagnosis to a certain extent. However, clinical manifestations of AE differ in patients form different countries and regions, as well as in patients with AE subtypes. The applicability of this diagnostic criterion in Chinese patients is not yet known, and large studies are needed to verify results. The relationships between antibody titers, disease severity and prognosis have not yet been clarified. Previous studies suggested that the correlation between antibody titers and prognosis is not obvious. Studies performed in other countries revealed that serum NMDAR antibodies can exist in a variety of diseases, such as stroke, Parkinson's disease, and others (25–27). Antibody testing patients diagnosed with AE according to the diagnostic criteria of Graus et al. in 2016 may be negative (23). In our cohort, there was no significant difference observed regarding antibody titer between good- and poor-prognosis groups. Further, in some patients, positive antibodies in serum or CSF were detectable after patients were in remission 6 months (data not shown). Therefore, the antibody test results should be considered rationally. The relationship between AE antibody titer and disease severity should be dynamically reviewed in a study that includes a large number of patients.

In this study, we analyzed factors that were related to poor AE prognosis, which revealed that older patients tended to have a poor prognosis, and is consistent with previous research. In addition, we found that patients with mental behavioral disorders, movement disorder, disturbance of consciousness, central hypoventilation, and those with tumors were overrepresented in the poor-prognosis vs. good-prognosis group. This provides a basis for us to evaluate early-stage AE prognosis. In particular, it should be noted that 13 (7.03%) patients with impaired consciousness and central hypoventilation were admitted to the ICU due to SE or respiratory failure. This suggests that attention should be paid to the poor prognosis of such patients. Previous studies of prognostic risk factors of AE reported similar findings (28). Mo et al. revealed that age, disturbance of consciousness, and ≥ 50% slow waves on EEG were independent risk factors of a poor AE prognosis (29). In addition, the proportion of abnormal brain MRI signals in the poor-prognosis group was significantly higher than that of the good-prognosis group, which was consistent with prior studies. Findings of Qiu et al. suggested that low serum albumin, consciousness disorders, epileptic seizures, central hypoventilation, and pulmonary infection complications were associated with a poor AE prognosis (30). It should be noted that of all 185 AE patients included in the study cohort, only 95 (51.35%) patients had abnormal brain MRI signals. Patients with clinical symptoms consistent with the diagnosis of AE may not display abnormal brain MRI signals. This study preliminarily assessed prognostic factors of AE. Due to the limited sample size, construction of a prognostic evaluation model was not possible. Additional limitations may include the retrospective nature of the study which may allow for selection bias. Therefore, the size of the cohort will be expanded in future studies.

## Conclusion

In summary, clinical characteristics, treatment, clinical outcomes, and prognostic factors in AE were assessed. We found that advanced age, and the presence of mental behavioral disorders, movement disorder, disturbance of consciousness, central hypoventilation, and tumors increased the likelihood having a poor AE prognosis. Moreover, use of steroids and IVIG combined immunotherapy tended to improve the prognosis of AE patients. Due to the limited number of participants included in the study, more diagnostic factors will likely be identified. Further exploration of prognostic factors and validation of study findings may be accomplished in the future by conducting a prospective, large-scale, randomized, controlled trial.

## Data Availability Statement

The original contributions generated for the study are included in the article/supplementary material, further inquiries can be directed to the corresponding author/s.

## Ethics Statement

The studies involving human participants were reviewed and approved by the Ethics Committee of Qilu Hospital of Shandong University. Written informed consent to participate in this study was provided by the participants' legal guardian/next of kin.

## Author Contributions

X-wL and S-cZ conceived the study, supervised the work, participated in its design and coordination. SQ conceived the study, collect data, organize and statistical data, and drafted the manuscript. H-kW, L-lL, R-rZ, and M-lW assisted in collecting data. TH assisted in statistics and organize data. All authors read and approved the final version of the manuscript.

## Conflict of Interest

The authors declare that the research was conducted in the absence of any commercial or financial relationships that could be construed as a potential conflict of interest.
